# Phytochemical Characterization and Evaluation of Antioxidant, Anti-Inflammatory, Cytotoxic, Genotoxic, and Anti-Arthritic Activities of *Atriplex halimus* Aqueous Leaf Extract

**DOI:** 10.3390/plants15142164

**Published:** 2026-07-14

**Authors:** Khalil Kaouane, Soraya Madoui, Hamza Kemchache, Hanane Khither, Amina Safsaf, Khalida Hammoudi, Stefania Ponticelli, Martina Dentato, Alessia Postiglione, Chawki Bensouici, Daniela Rigano, Carmina Sirignano, Viviana Maresca

**Affiliations:** 1 Laboratory of Applied Biochemistry, Faculty of Nature and Life Sciences, Ferhat Abbas Setif-1-University, Setif 19000, Algeria; khalil.kaouane@univ-setif.dz (K.K.); madoui.soraya@univ-setif.dz (S.M.); hamza.kemchache@univ-setif.dz (H.K.); khither.hanane@univ-setif.dz (H.K.); 2 Pathology Laboratory, Setif University Hospital, Setif 19000, Algeria; aminasafsaf1984@gmail.com; 3 Faculty of Medicine, Ferhat Abbas Setif-1-University, Setif 19000, Algeria; hammoudi.k85@gmail.com; 4 Department of Biology, University of Naples Federico II, Via Cinthia 4, 80126 Napoli, Italy; ste.ponticelli@studenti.unina.it (S.P.); martina.dentato@unina.it (M.D.); alessia.postiglione@unina.it (A.P.); 5 National Center for Biotechnology Research, Ali Mendjli, Nouvelle Ville UV 03, Constantine 25011, Algeria; c.bensouici@crbt.dz; 6 Department of Pharmacy, School of Medicine and Surgery, University of Naples Federico II, Via Domenico Montesano 49, 80131 Naples, Italy; daniela.rigano@unina.it (D.R.); carmina.sirignano@unina.it (C.S.); 7 Department of Life Sciences, Health and Health Professions, University of Rome “Link Campus”, 00165 Rome, Italy

**Keywords:** *Atriplex halimus*, antioxidant, anti-inflammatory, cytotoxic, genotoxic, anti-arthritic

## Abstract

*Atriplex halimus* is a medicinal plant traditionally used for various therapeutic purposes. This study evaluated the antioxidant, anti-inflammatory, cytotoxic, genotoxic, and anti-arthritic activities of the *A. halimus* aqueous extract (AHA). Anti-arthritic effects were investigated in Complete Freund’s Adjuvant (CFA)-induced arthritic rats treated with AHA (150 or 300 mg/kg) for 21 days. AHA significantly reduced paw swelling and arthritis severity and improved body weight, while a non-significant reduction in spleen enlargement was observed. Hematological and biochemical parameters were restored toward normal values, indicating anti-inflammatory, hepatoprotective, and nephroprotective effects. The extract also reduced oxidative stress by decreasing nitric oxide (NO) and malondialdehyde (MDA) levels and increasing glutathione (GSH) content and catalase (CAT) activity. Histopathological examination confirmed reduced inflammatory infiltration and protection against bone damage. Phytochemical analysis revealed polyphenols, flavonoids, and tannins, consistent with its antioxidant activity in DPPH, ABTS, CUPRAC, and *o*-phenanthroline assays. AHA also showed anti-inflammatory activity by inhibiting bovine serum albumin denaturation. The extract induced a moderate concentration-dependent reduction in HeLa cell viability without cytotoxicity toward HaCaT cells. Comet assay results demonstrated DNA damage in HeLa cells at higher concentrations, while AHA significantly suppressed zymosan-induced IL-1β gene expression. These findings indicate that AHA is a promising natural source of bioactive compounds with diverse biological activities.

## 1. Introduction

Oxidative stress, the imbalance between reactive oxygen species (ROS) production and antioxidant defenses, is now widely regarded as a key factor in many chronic diseases [1]. For example, elevated ROS and downstream damage are implicated in cancer, cardiovascular disease, neurodegenerative disorders, and autoimmune conditions, including rheumatoid arthritis (RA). In RA, excessive ROS production from inflamed synovium and infiltrating immune cells damages lipids, proteins, and DNA, fueling inflammation and tissue injury [2,3].

In RA patients (and animal models), markers of oxidative damage are consistently elevated, and antioxidant defenses are impaired. For instance, levels of malondialdehyde (MDA), one of the major end-products of lipid peroxidation, are significantly higher in RA sera and synovial fluid [4]. Likewise, markers of DNA oxidation (e.g., 8-hydroxydeoxyguanosine) are elevated, and nitric oxide (NO), a reactive nitrogen species, is overproduced in RA joints and blood [5]. By contrast, enzymatic antioxidants are depleted: activities of superoxide dismutase (SOD), catalase (CAT), and glutathione peroxidase (GPx) are markedly lower in RA patients than in healthy controls. Similarly, levels of reduced glutathione (GSH), a key endogenous antioxidant, are diminished in RA synovial tissue and serum [6]. These changes create a vicious cycle: accumulated ROS amplify synovial inflammation and cartilage/bone destruction, which in turn further increases oxidative damage [7].

Because oxidative stress perpetuates RA, treatments that combine anti-inflammatory action with redox support are gaining interest. Recent reviews have noted that adjunctive antioxidant therapies can reduce both systemic and local oxidative stress, thereby ameliorating tissue damage in autoimmune arthritis [8]. In fact, recent meta-analyses and controlled studies indicate that antioxidant supplementation, ranging from vitamins such as C and E to coenzyme Q10 and plant-derived polyphenols, can modestly reduce inflammatory markers while enhancing antioxidant enzyme activity in patients with rheumatoid arthritis [9,10,11].

Plant-derived polyphenols and flavonoids are especially promising adjuncts. These compounds (found in fruits, vegetables, herbs, and spices) have well-documented antioxidant, anti-inflammatory, and immunomodulatory effects [12]. Structurally, flavonoids can chelate metal ions and terminate ROS (for example, scavenging hydroxyl radicals) [13]. They also downregulate pro-inflammatory cytokines (e.g., IL-1β, IL-6, TNF-α) and boost endogenous antioxidant defenses [14]. Experimental and clinical studies show polyphenol-rich botanicals (e.g., curcumin, resveratrol, and quercetin) reduce oxidative markers and improve immune balance in models of RA and other inflammatory diseases [15,16]. One plant of particular interest is *Atriplex halimus* L. This halophytic shrub is widely distributed in Mediterranean and North African regions and has been used in traditional medicine for generations. Its leaves are prescribed for ailments ranging from chest and digestive disorders to diabetes, cardiovascular problems, and rheumatic pain [17]. Modern phytochemical studies confirm that *A. halimus* contains significant amounts of phenolic acids and flavonoids, supporting its traditional use [18,19].

Despite these indications, experimental validation of the anti-arthritic properties of *A. halimus* remains limited. In addition to their antioxidant and anti-inflammatory activities, plant-derived polyphenols may exert important effects on cellular viability, genomic stability, and inflammatory gene regulation. Several studies have demonstrated that phenolic-rich extracts can selectively inhibit tumor cell proliferation while preserving normal cell viability through modulation of oxidative stress, apoptosis, and inflammatory signaling pathways [20,21]. Moreover, chronic inflammation and excessive reactive oxygen species production are closely associated with DNA damage and activation of pro-inflammatory mediators such as interleukin-1 beta (IL-1β), a key cytokine involved in inflammatory and autoimmune disorders [22]. Therefore, the evaluation of cytotoxicity, genotoxicity, and inflammatory gene expression represents an important approach for assessing both the biological potential and safety profile of medicinal plant extracts.

In this context, the present study investigated the antioxidant, anti-inflammatory, cytotoxic, genotoxic, and anti-arthritic activities of the aqueous extract of *Atriplex halimus*. The effects of the extract on cellular viability were evaluated in HeLa and HaCaT cell lines, while DNA damage was assessed using the alkaline Comet assay. In addition, the immunomodulatory potential of the extract was explored through the analysis of zymosan-induced IL-1β gene expression in HaCaT cells. Antioxidant activity was assessed in vitro using DPPH, ABTS, CUPRAC, and *o*-phenanthroline assays, whereas anti-inflammatory activity was evaluated through inhibition of protein denaturation. The anti-arthritic potential was further examined in vivo using a Complete Freund’s Adjuvant (CFA)-induced arthritis rat model. Disease progression was monitored through paw and joint swelling, body weight, spleen index, hematological and biochemical parameters, oxidative stress biomarkers (MDA, NO, CAT, and GSH), and histopathological evaluation of the arthritic paw tissue. By integrating in vitro and in vivo approaches, this study aimed to provide scientific evidence supporting the traditional use of *A. halimus* in rheumatic disorders and to explore its potential as a plant-based therapeutic agent targeting inflammation, oxidative stress, and inflammatory signaling pathways.

## 2. Results

### 2.1. Total Phenolic (TPC), Flavonoids (TFC), and Condensed Tannins Content (CTC)

The phytochemical analysis of AHA extract showed that the TPC was 15.73 ± 1.71 µg Gallic Acid Equivalent (GAE)/mg extract, the TFC was 4.73 ± 0.77 µg Quercetin Equivalent (QE)/mg extract, and the CTC was 2.10 ± 0.59 µg Catechin Equivalent (CE)/mg extract.

### 2.2. LC-ESI/HRMS Characterization of Atriplex halimus Aqueous Extract

The LC-ESI/HRMS analysis of the aqueous extract of *Atriplex halimus* allowed the tentative identification of 30 metabolites belonging to different chemical classes, including flavonoids, hydroxycinnamic acid derivatives, phenolic acids, amino acids and their derivatives, oxylipins, organic acids, and minor polar metabolites. The identification was achieved based on accurate mass measurements, MS/MS fragmentation patterns, retention times, and comparison with literature data.

Among the detected compounds (Table 1 and Table 2), flavonol glycosides represented one of the most structurally complex classes. In particular, several highly glycosylated flavonols were identified, including compound **9′**, tentatively assigned as an isorhamnetin pentasaccharide, compound **10′**, assigned as a syringetin trisaccharide, and compounds **11′**, **13′** and **16′**, attributed to atriplexoside-type flavonols, namely atriplexoside B or isorhamnetin tetrasaccharide I (**11′**), atriplexoside B or isorhamnetin tetrasaccharide II (**13′**), and atriplexoside A or isorhamnetin trisaccharide (**16′**).

The occurrence of diagnostic aglycone-related fragment ions allowed the differentiation of methoxylated flavonol cores. In particular, compound **10′** showed characteristic fragment ions at *m*/*z* 345.0600, 330.0368, and 315.0514, supporting the presence of a syringetin nucleus. The ion at *m*/*z* 345 corresponds to the deprotonated syringetin aglycone, while the ions at *m*/*z* 330 and 315 can be attributed to sequential demethylation processes, which are typical of dimethoxylated flavonols [23]. In contrast, compounds **9′**, **11′**, **13′**, and **16′** displayed diagnostic fragment ions at *m*/*z* 315 and 314, corresponding to the deprotonated and radical forms of the isorhamnetin aglycone, respectively. These fragmentation patterns are consistent with monomethoxylated flavonols and confirm the presence of isorhamnetin-derived glycosides. Compound **9′** (*m*/*z* 1019.28790) showed a neutral loss of 132 Da, indicating the presence of a pentosyl unit, while compounds **11′** and **13′** (*m*/*z* 887.2465) exhibited fragment ions at *m*/*z* 755.2040, corresponding to the loss of a hexosyl residue. Compound **16′** (*m*/*z* 755.20490) showed fragmentation consistent with a triglycosylated flavonol. Overall, these findings indicate that flavonols in the aqueous extract occur predominantly as highly glycosylated methoxylated derivatives, structurally related to atriplexoside-type compounds previously reported in *Atriplex halimus* [24].

The hydroxycinnamic acid derivatives included both sulfated phenolic acids and organic acid conjugates. Compounds **2′** and **4′** were identified as positional isomers of caffeic acid sulfate based on the diagnostic fragment ions at *m*/*z* 96.96 and 79.96 (HSO_4_^−^ and SO_3_^−^), together with the ion at *m*/*z* 179 corresponding to deprotonated caffeic acid. In addition, compounds **12′**, **14′**, and **15′** were assigned as *p*-coumaroyl- and feruloyl-isocitric acid derivatives, based on characteristic fragment ions at *m*/*z* 191, 173, and 111, which are indicative of the isocitric acid moiety and allow their differentiation from structurally related quinic acid conjugates [25]. Overall, the occurrence of both flavonol glycosides and hydroxycinnamic acid derivatives is in agreement with previous phytochemical studies on *Atriplex halimus*, further supporting the reliability of the present LC–MS/MS-based annotations.

Phenolic acids and benzoic acid derivatives, including gentisic acid (**3′**) [26] and 3-hydroxybenzoic acid (**17′**) [27], were characterized by typical decarboxylation pathways leading to fragment ions corresponding to the loss of CO_2_. The presence of caffeic acid (**5′**) was supported by the diagnostic fragment at *m*/*z* 135. Additionally, 4-hydroxyquinoline-2-carboxylic acid (**1′**) contributed to the diversity of aromatic metabolites detected [28].

The extract also contained several amino acids and related metabolites, including L-tyrosine (**4**), L-phenylalanine (**6**), and L-tryptophan (**7**) [29,30], together with their derivatives such as *N*-acetylphenylalanine (**9**), *N*-acetyltryptophan (**10**), 3-phenyllactic acid (**8**), and 3-(*N*-sulfonylindolyl)-D-lactic acid (**6′**) [31,32]. Their fragmentation patterns, including neutral losses of NH_3_ and CO_2_, were consistent with typical amino acid behavior in negative ion mode. The simultaneous occurrence of these compounds supports the presence of an active shikimate and phenylpropanoid pathway, linking primary and secondary metabolism.

Among lipid-derived metabolites, two oxylipins, namely 12-hydroxyjasmonic acid (**8′**) and azelaic acid (**12**), were identified. These compounds are associated with plant stress signaling and oxidative responses, and their presence is consistent with the adaptive metabolism of *A. halimus*, a halophytic species exposed to harsh environmental conditions [33]. Furthermore, several organic acids related to the tricarboxylic acid (TCA) cycle, including isocitric acid (**1**), citric acid (**2**), aconitic acid (**11**), and tricarballylic acid (**5**), were detected. Their fragmentation patterns, characterized by dehydration and decarboxylation processes, are typical of polycarboxylic acids analyzed in negative ion mode and reflect the abundance of primary metabolic intermediates in the aqueous extract [34,35].

Finally, minor metabolites such as a sulfated derivative (**7′**) and a tentatively identified gingerol-like compound (**18′**) were detected, further contributing to the chemical diversity of the extract. Overall, the phytochemical profile of the aqueous extract of *Atriplex halimus* provides a strong chemical basis for the anti-inflammatory, antioxidant, and anti-arthritic activities observed in the biological assays. The high abundance of flavonol glycosides, including isorhamnetin- and syringetin-derived compounds (**9′**–**11′**, **13′**, **16′**), is particularly relevant, as these metabolites are well known for their antioxidant and anti-inflammatory properties. Methoxylated flavonols such as isorhamnetin and syringetin have been reported to modulate inflammatory pathways by inhibiting pro-inflammatory mediators and reducing oxidative stress [36]. In addition, the presence of phenolic acids and hydroxycinnamic acid derivatives, such as caffeic acid and its sulfated forms (**2′**, **4′**, **5′**), further supports the antioxidant potential of the extract. These compounds are known to act as free radical scavengers and to inhibit lipid peroxidation, which is a key mechanism in inflammatory and arthritic conditions [37].

**Table 1:** Primary metabolites identified in the aqueous extract of *A. halimus*.

No.	Name	R*_t_*	Delta ppm	Molecular Formula	[M-H]^−^	MS/MS	Ref.
**1**	isocitric acid	1.02	5.14	C_6_H_8_O_7_	191.0196	173.0093, 154.9989, 147.0298, 129.0195, 111.0088, 85.0296, 72.9932	[33]
**2**	citric acid	1.17	5.29	C_6_H_8_O_7_	191.0196	173.0093, 154.9989, 147.0293, 129.0195, 111.0088, 85.0296	[33]
**3**	hypoxanthine	1.24	9.05	C_5_H_4_ON_4_	135.0314	92.0255, 65.0146	[38]
**4**	L-tyrosine	1.28	6.55	C_9_H_11_O_3_N	180.0667	136.0770, 90.9860	[28]
**5**	tricarballylic acid	1.31	6.43	C_6_H_8_O_6_	175.0248	146.9614, 118.9665, 44.9983	[34]
**6**	L-phenylalanine	1.82	6.86	C_9_H_11_O_2_N	164.0717	103.0554	[28]
**7**	L-tryptophan	3.63	5.30	C_11_H_12_O_2_N_2_	203.0826	159.0929, 142.0664, 116.0507, 74.0248	[29]
**8**	3-phenyllactic acid	5.97	6.96	C_9_H_10_O_3_	165.0558	147.0453, 119.0504, 72.9932	[31]
**9**	*N*-acetylphenylalanine	6.09	4.99	C_11_H_13_O_3_N	206.0822	148.0453, 103.0554, 91.0554	[30]
**10**	*N*-acetyltryptophan	6.74	3.92	C_13_H_14_O_3_ N_2_	245.0930	186.0926, 116.0354	[30]
**11**	aconitic acid	6.80	6.22	C_6_H_6_O_6_	173.0091	154.9988, 129.0196, 111.0089, 85.0296	[39]
**12**	azelaic acid	7.47	5.69	C_9_H_16_O_4_	187.0975	169.0871, 143.1079, 125.0973, 97.0659	[32]

**Table 2:** Specialized metabolites identified in the aqueous extract of *A. halimus*.

No.	Name	R*_t_*	Delta ppm	Molecular Formula	[M-H]^−^	MS/MS	Ref.
**1′**	4-hydroxyquinoline-2-carboxylic acid	4.80	6.12	C_10_H_7_O_3_N	188.0354	144.0456	[27]
**2′**	*trans*-caffeic acid 4-sulfate	4.86	4.44	C_9_H_8_O_7_S	258.99185	179.0349, 135.0453, 96.9602, 79.9574	[24]
**3′**	gentisic acid	5.00	7.35	C_7_H_6_O_4_	153.01942	135.0090, 109.0296, 91.0190	[25]
**4′**	*trans*-caffeic acid 3-sulfate	5.17	4.21	C_9_H_8_O_7_S	258.9918	179.0349, 135.0453, 96.9602, 79.9574	[24]
**5′**	caffeic acid	5.18	6.67	C_9_H_8_O_4_	179.03508	135.0300	[37]
**6′**	3-(*N*-sulfonylindolyl)-*D*-lactic acid	5.25	4.31	C_11_H_11_O_6_NS	284.0235	266.0135, 222.0232, 204.0667, 142.0664, 96.9602, 79.9574	[24]
**7′**	sulphated derivative	5.93	1.20	C_14_H_26_OS_3_	305.1066	96.9601, 79.9573	[38]
**8′**	12-hydroxyjasmonic acid	5.95	5.08	C_12_H_18_O_4_	225.1133	147.0818, 59.0139	[37]
**9′**	isorhamnetin pentasaccharide	6.40	0.45	C_43_H_56_O_28_	1019.2879	887.2457, 314.0432, 315.0505, 299.0198	[24]
**10′**	syringetin trisaccharide	6.45	2.59	C_34_H_42_O_21_	785.2149	653.1727, 345.0600, 330.0368, 315.0514	[24]
**11′**	atriplexoside B or isorhamnetin tetrasaccharide I	6.52	1.34	C_38_H_48_O_24_	887.2465	755.2040, 315.0502, 314.0432, 299.0197	[24]
**12′**	*p*-coumaroylisocitric acid	6.56	2.94	C_15_H_14_O_9_	337.0564	191.0198, 173.0092, 154.9987, 111.0088	[24]
**13′**	atriplexoside B or isorhamnetin tetrasaccharide II	6.69	1.34	C_38_H_48_O_24_	887.2465	755.2040, 315.0502, 314.0432, 299.0197	[24]
**14′**	feruloylisocitric acid I	6.79	2.09	C_16_H_16_O_10_	367.0669	191.0196, 173.0092, 154.9987, 111.0088	[24]
**15′**	feruloylisocitric acid II	6.95	2.09	C_16_H_16_O_10_	367.0669	191.0196, 173.0092, 154.9987, 111.0088	[24]
**16′**	atriplexoside A or isorhamnetin trisaccharide	7.10	1.98	C_33_H_40_O_20_	755.20490	315.0510, 314.0434, 300.0276, 299.0199, 271.0250	[24]
**17′**	3-hydroxybenzoic acid	7.19	8.46	C_7_H_6_O_3_	137.0245	93.0346	[27]
**18′**	putative gingerol isomer	11.07	3.77	C_17_H_26_O_4_	293.1758	236.1054, 221.1547, 220.1469, 193.1242	[40]
**19′**	unidentified compound	12.97	6.35	C_18_H_20_ON	265.1479	96.9601	
**20′**	unidentified compound	15.98	2.87	C_21_H_28_O_2_	311.2015	149.0973	

### 2.3. In Vitro Antioxidant Activity

Antioxidant activity was expressed as IC_50_ values for the DPPH and ABTS assays and as A_0.5_ values for the CUPRAC and *o*-phenanthroline assays. In all tests, lower IC_50_ values indicated stronger antioxidant activity (Table 3). The recorded values ranged from 136.68 ± 1.55 to 697.77 ± 2.08 µg/mL. The extract showed the highest antioxidant activity in the phenanthroline assay (136.68 ± 1.55 µg/mL) and the lowest activity in the DPPH assay (697.77 ± 2.08 µg/mL). Overall, the antioxidant effectiveness followed the order: *o*-phenanthroline > ABTS > CUPRAC > DPPH. Regarding the reference antioxidants, BHA exhibited the greatest activity in the DPPH, CUPRAC, and *o*-phenanthroline assays, whereas BHT showed superior activity in the ABTS assay, with an IC_50_ value of 1.29 ± 0.30 µg/mL.

### 2.4. In Vitro Anti-Inflammatory Activity

In the present study, AHA exhibited an IC_50_ value of 84.31 ± 2.42 µg/mL (*p* < 0.01) in the BSA denaturation assay, which is comparable to diclofenac sodium (IC_50_ = 75.57 ± 0.83 µg/mL). Although diclofenac showed slightly greater inhibitory activity, the extract demonstrated substantial protection against heat-induced protein denaturation.

### 2.5. In Vitro Cytotoxicity Activity

**Figure 1:**
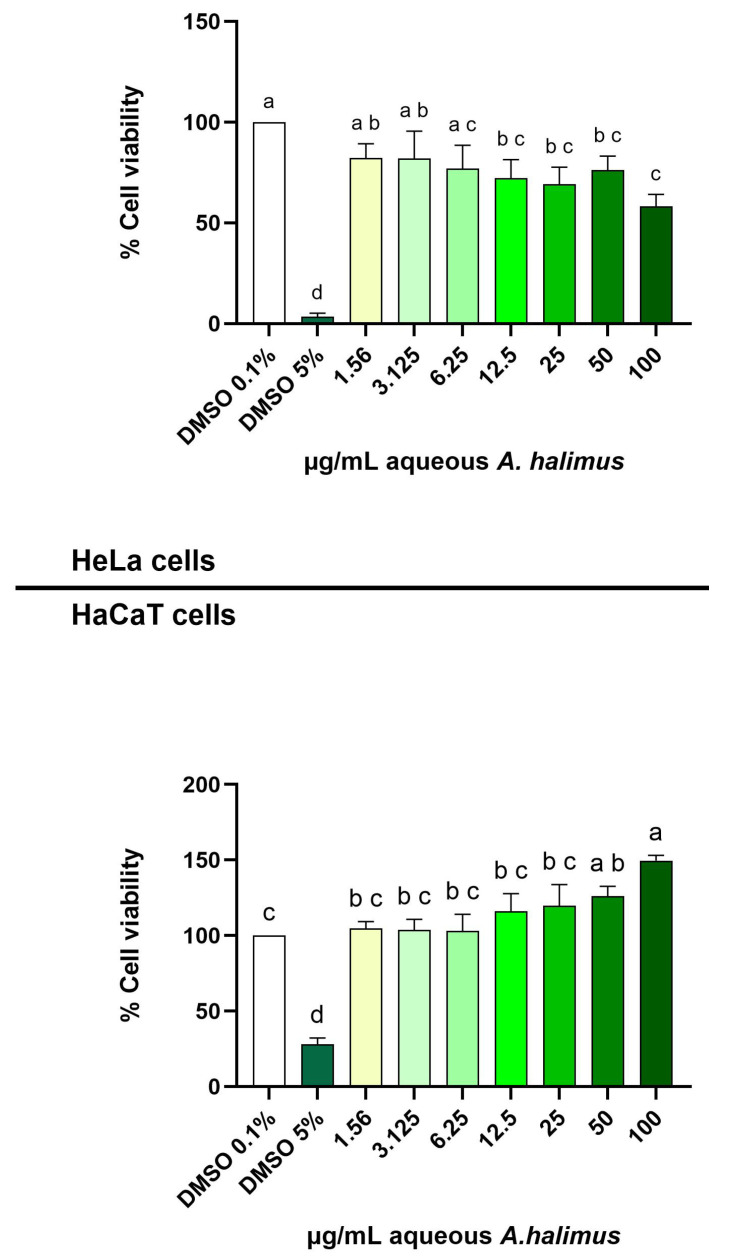
Effect of the AHA on cell viability of HeLa and HaCaT cells at different concentrations (1.56, 3.125, 6.25, 12.5, 25, 50, 100 µg/mL) after 24 h of treatment. Negative control was represented by DMSO 0.1 %. Positive control was represented by DMSO 5%. Data are presented as mean ± SD (n = 3). Statistical significance was determined by one-way ANOVA followed by Tukey’s post-hoc test. Different letters indicate statistically significant differences among treatments (*p* < 0.05).

AHA, as shown in Figure 1, exhibited a differential effect on cell viability in human cervical cancer cells (HeLa) and non-tumorigenic human keratinocytes (HaCaT). Specifically, AHA induced a moderate reduction in viability in HeLa cells, while displaying no cytotoxic effects, and even a stimulatory response toward HaCaT cells. This distinct response between the two cell lines suggests a degree of selective biological activity, which is considered an important feature for compounds with potential therapeutic applications.

The results obtained from the cytotoxicity assay in HeLa cells revealed a concentration-dependent decrease in cell viability following exposure to AHA. At lower and intermediate concentrations (1.56–50 µg/mL), a modest reduction in viability was observed, with values generally ranging between approximately 70% and 85% compared to the control. However, at the highest tested concentration (100 µg/mL), a more pronounced decrease in viability was detected, reaching approximately 60%. Although the overall effect was moderate, these findings indicate that AHA exerts a dose-dependent cytotoxic action on cancer cells.

In contrast, HaCaT cells exhibited a markedly different response profile. While treatment with 5% DMSO significantly reduced cell viability, exposure to AHA did not induce cytotoxicity at any of the tested concentrations. Instead, a progressive increase in cell viability was observed with increasing concentrations of the extract. Notably, at higher concentrations (50 and 100 µg/mL), cell viability exceeded 120–140% relative to the control, suggesting a potential stimulatory or cytoprotective effect on non-tumorigenic cells.

Overall, these findings demonstrate that the AHA exerts a moderate cytotoxic effect on HeLa cells while preserving, and potentially enhancing, the viability of HaCaT cells. This differential response supports the hypothesis of selective activity toward cancer cells and is consistent with previous reports indicating that plant-derived extracts may preferentially target malignant cells while sparing normal tissues.

### 2.6. In Vitro Genotoxicity Activity

**Figure 2:**
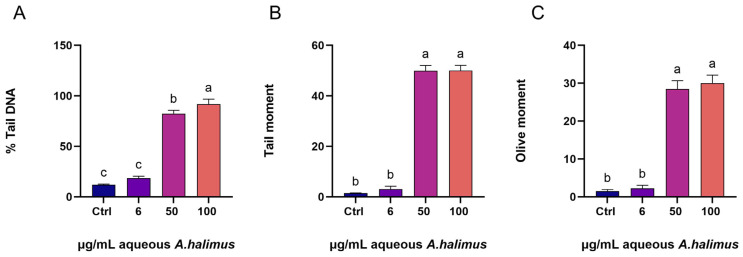
Genotoxicity assessment of AHA in HeLa cells using the alkaline Comet assay. Cells were treated with increasing concentrations of the AHA (6, 50, and 100 µg/mL) for 24 h. (**A**) The percentage of tail DNA, (**B**) tail moment, and (**C**) Olive tail moment were quantified to evaluate DNA damage. Data are presented as mean ± SD (n = 3). Statistical significance was determined by one-way ANOVA followed by Tukey’s post-hoc test. Different letters indicate statistically significant differences among treatments (*p* < 0.05).

The genotoxic potential of AHA was evaluated using the alkaline Comet assay. As shown in Figure 2, treatment with increasing concentrations of the extract (6, 50, and 100 µg/mL) induced a concentration-dependent increase in DNA strand breaks, as evidenced by significant changes in key Comet parameters.

Specifically, the percentage of tail DNA increased significantly at 50 and 100 µg/mL compared to the control, indicating enhanced DNA fragmentation (Figure 2A). A similar trend was observed for the tail moment, which showed a marked increase at 50 and 100 µg/mL, reflecting a greater extent of DNA migration (Figure 2B). Likewise, the Olive moment was significantly elevated at the same concentrations (*p* < 0.05), further confirming the induction of DNA damage (Figure 2C). In contrast, treatment at 6 µg/mL did not result in significant alterations in any of the evaluated parameters.

Fluorescence microscopy images (Olympus BX53, Olympus Corporation, Tokyo, Japan) of the Comet assay are shown in Supplementary Materials (Figure S1), illustrating control cells and treated cells, with a progressive increase in comet tail formation observed at higher concentrations.

The AHA induces measurable genotoxic effects at higher concentrations, whereas lower concentrations appear to have minimal impact on DNA integrity.

### 2.7. Effect of Extract Pretreatment on IL-1β Gene Expression

**Figure 3:**
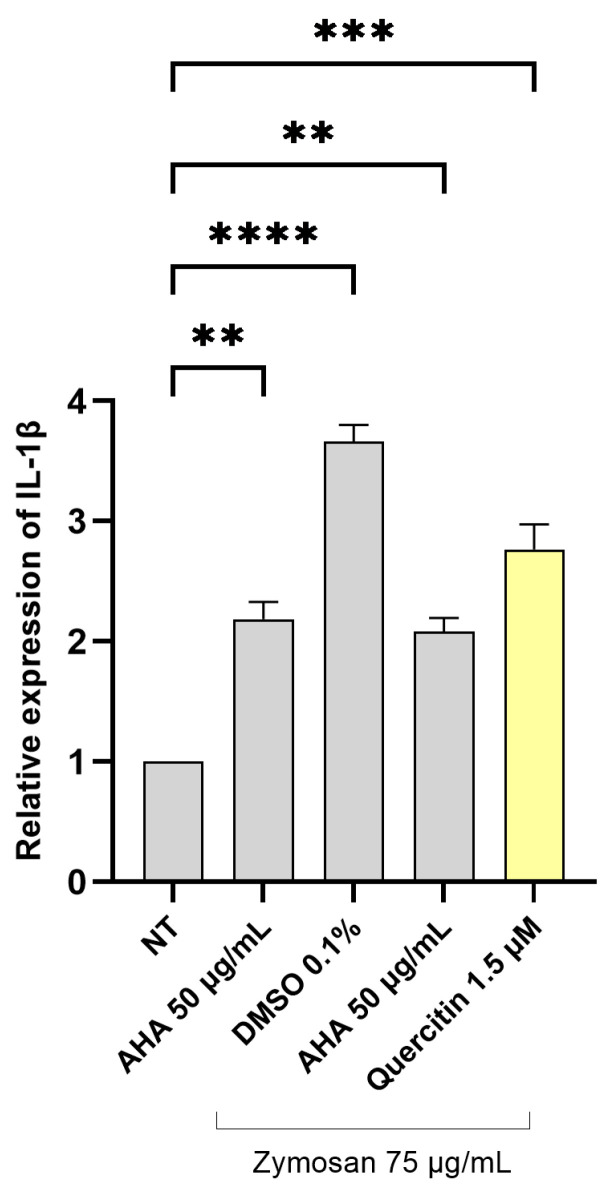
Relative mRNA expression of IL-1β normalized to GAPDH as endogenous control. Cells were left untreated (NT), treated with AHA (50 μg/mL) alone, or pre-treated with vehicle (DMSO 0.1%), AHA (50 μg/mL), or quercetin (1.5 μM) for 24 h before stimulation with zymosan (75 μg/mL), where indicated. Data are expressed as fold change relative to the non-treated control (NT = 1.0) and shown as mean ± SD (n = 3). Statistical analysis was performed by one-way ANOVA with post hoc tests comparing all experimental conditions against the NT group (** *p*-value < 0.01, *** *p*-value < 0.001, **** *p*-value < 0.0001).

Stimulation with zymosan (75 μg/mL) induced a strong increase in IL-1β mRNA expression (Figure 3). In the vehicle control group (DMSO 0.1%), zymosan stimulation resulted in an approximately 3.7-fold upregulation of IL-1β compared to the non-treated control (NT = 1.0).

Treatment with AHA (50 μg/mL) alone (without zymosan) caused a moderate increase in IL-1β expression, reaching approximately 2.2-fold relative to NT. Although AHA alone induced a moderate increase in basal IL-1β expression, this effect was markedly lower than that elicited by zymosan stimulation and did not prevent the extract from significantly attenuating the inflammatory response following zymosan challenge. This context-dependent pattern is consistent with a hormetic-like response, which is discussed further below. However, when cells were pre-treated with AHA prior to zymosan stimulation, IL-1β mRNA levels were significantly reduced to 2.1-fold, demonstrating a clear inhibitory effect on zymosan-induced IL-1β expression (*p* < 0.0001 compared to DMSO + zymosan).

The positive control quercetin (1.5 μM) also attenuated the zymosan-induced response, lowering IL-1β expression to approximately 2.8-fold relative to NT. This reduction was statistically significant compared to the vehicle control but less effective than the inhibition observed with AHA.

The AHA effectively counteracted zymosan-triggered IL-1β upregulation, bringing expression levels close to those observed with AHA treatment alone and significantly below the vehicle-stimulated condition.

The dual effect (pro-inflammatory alone, anti-inflammatory under strong stimulation) is consistent with hormesis, a biphasic response where a substance induces opposite effects depending on the context or dose. This phenomenon is well documented in plant extracts, where 70–80% display such biphasic immune modulation [41].

### 2.8. In Vivo Antiarthritic Activity

#### 2.8.1. Measurement of Body Weight

**Figure 4:**
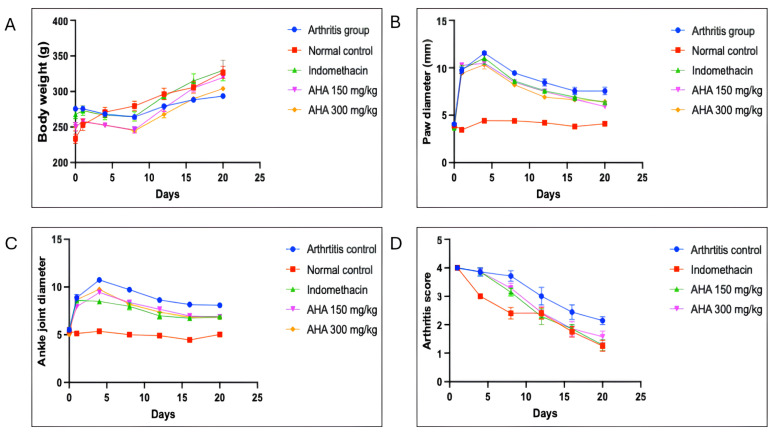
Effect of the aqueous extract on body weight (**A**), paw diameter (**B**), ankle joint diameter (**C**), and arthritis score (**D**) in complete Freund’s adjuvant (CFA)-induced arthritis in rats. Values are expressed as mean ± SEM (n = 7). Statistical analysis was performed using two-way ANOVA followed by Dunnett’s post hoc test.

A substantial (*p* < 0.001) reduction in body weight was observed in all rats treated with CFA compared to the normal control group. On the 21st day, treatment with AHA at doses of 150 mg/kg and 300 mg/kg resulted in a significant increase in body weight, reaching 319.76 ± 2.12 g and 304.13 ± 2.68 g, respectively. In contrast, the indomethacin-treated group exhibited an increase in body weight of 328.07 ± 14.6 g, while the arthritis control group maintained a body weight of 293.56 ± 3.05. Notably, the AHA group at doses of 150 and 300 mg/kg began gaining weight from day 8th onwards (Figure 4).

#### 2.8.2. Measurement of Paw and Ankle Joint Diameter

**Figure 5:**
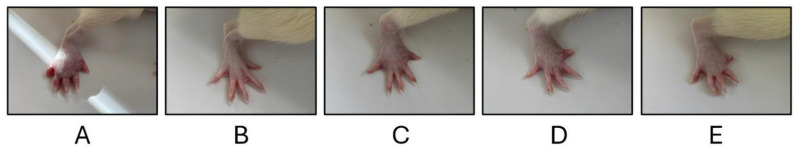
Effects of aqueous extract of *A halimus* on paw diameter against CFA-induced arthritis on day 21. (**A**) CFA control; (**B**) normal control; (**C**) standard drug Indomethacin; (**D**,**E**) treatment with 150, 300 mg/kg extract, respectively.

There was a significant increase (*p* < 0.0001) in paw and joint diameters in rats from all CFA-treated groups compared with the normal control group (Figure 4). A significant decrease (*p* < 0.01) in both paw and joint diameters was observed in the groups treated with indomethacin (5 mg/kg) and AHA (150 and 300 mg/kg) on the 21st day compared with the arthritic control group. Moreover, redness and swelling of the affected joints were markedly reduced in treated animals compared with arthritic control animals (Figure 5).

#### 2.8.3. Measurement of Arthritic Score

On the 21st day, the CFA model revealed that the arthritis control group had a higher arthritis score. Treated groups with AHA (150 and 300 mg/kg) and indomethacin (5 mg/kg) showed a substantial (*p* ≤ 0.05) decrease in the arthritic index on the 21st day compared with the arthritic control rats, as shown in Figure 4).

#### 2.8.4. Measurement of Spleen Weight

**Figure 6:**
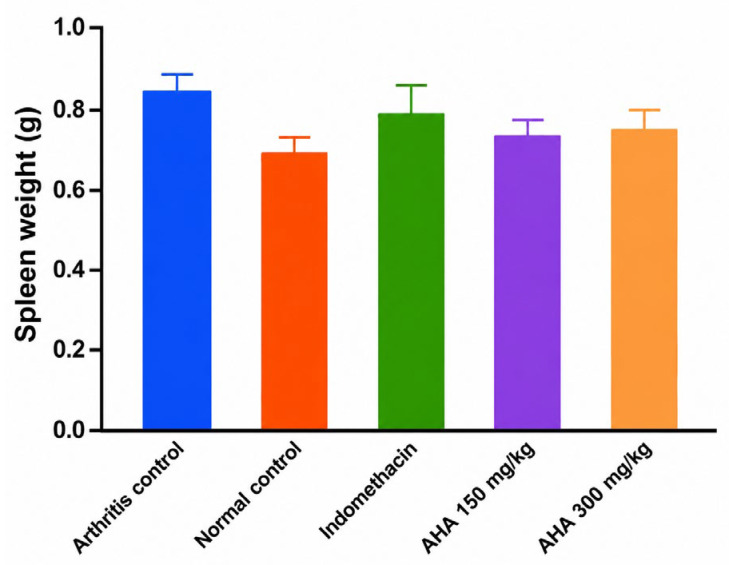
Effect of AHA extract on spleen weight in the arthritic rat model. Values are expressed as mean ± SEM (n = 7). Statistical analysis was performed using one-way ANOVA. Differences were considered non-significant.

On the 21st day of the study, spleen weight in the normal control group was 0.6896 ± 0.03 g. Administration of CFA increased spleen weight in the arthritic control group (0.805 ± 0.092 g). Treatment with AHA (150 mg/kg: 0.729 ± 0.03 g; 300 mg/kg: 0.74823 ± 0.04 g) and indomethacin (5 mg/kg: 0.7802 ± 0.03 g) non-significantly reduced spleen weight compared to the arthritic group (Figure 6).

#### 2.8.5. Measurement of Hematological Parameters

The arthritic control group showed reduced hemoglobin (Hb) content and red blood cell (RBC) counts compared to the normal control group, along with a non-significant elevation in white blood cell (WBC) count. A significant elevation in platelet (PLT) (*p* < 0.0001) counts was observed in arthritic rats as compared to normal control rats. AHA groups displayed a reduction in the levels (Table 4).

#### 2.8.6. Measurement of Biochemical Parameters

Serum biochemical parameters, including urea, creatinine, aspartate aminotransferase (AST), and alanine aminotransferase (ALT), were evaluated in the present study. Among these markers, only creatinine (*p* < 0.001) and ALT (*p* < 0.01) showed statistically significant increases in the arthritic control group compared with the normal control (Table 5). In contrast, the differences observed in Urea and AST levels were not statistically significant. Treatment with AHA extract at doses of 150 and 300 mg/kg significantly decreased creatinine levels compared to the arthritic control group, bringing them closer to normal values (Table 5).

#### 2.8.7. Measurement of Serum, Liver, and Spleen NO, GSH, MDA, and CAT

As depicted in Table 6, Table 7 and Table 8, CFA-induced arthritis altered oxidative stress biomarkers in serum, liver, and spleen tissues, as evidenced by changes in MDA, GSH, NO, and CAT levels compared with the normal control group. In serum (Table 6), the arthritic control group exhibited significantly elevated MDA levels and significantly reduced GSH levels compared with the normal control group (*p* < 0.001 and *p* < 0.01, respectively). In contrast, serum NO and CAT levels did not differ significantly between the two groups (ns). Treatment with AHA extract at both doses significantly reduced serum MDA levels (*p* < 0.001) and significantly increased GSH levels, with the 150 mg/kg dose producing a greater restoration (*p* < 0.01) than the 300 mg/kg dose (*p* < 0.05). Serum NO and CAT levels remained statistically unchanged relative to the arthritic control group.

In liver tissue (Table 7), CFA-induced arthritis resulted in significantly decreased GSH and CAT activities and significantly increased MDA and NO levels compared with the normal control group (*p* < 0.001). Treatment with AHA extract at both doses significantly reduced hepatic MDA and NO levels (*p* < 0.001). The 150 mg/kg dose significantly restored GSH content (*p* < 0.001) and CAT activity (*p* < 0.01), whereas the 300 mg/kg dose did not significantly improve GSH content or CAT activity relative to the arthritic control group.

In spleen tissue (Table 8), the arthritic control group showed significantly elevated MDA and NO levels together with significantly reduced GSH levels compared with the normal control group (*p* < 0.001). Although CAT activity was numerically lower in the arthritic control group, the difference was not statistically significant. Administration of AHA extract at both doses significantly reduced splenic MDA and NO levels (*p* < 0.001). Improvement in GSH content and CAT activity was also observed, with the 150 mg/kg dose producing significant increases in both GSH (*p* < 0.05) and CAT activity (*p* < 0.05), whereas the 300 mg/kg dose produced non-significant improvements.

Overall, the 150 mg/kg dose of AHA extract demonstrated greater efficacy than the 300 mg/kg dose in normalizing oxidative stress biomarkers across the examined tissues, suggesting a possible inverse dose–response relationship.

#### 2.8.8. Histopathological Examination of the Paw Tissue and Liver

**Figure 7:**
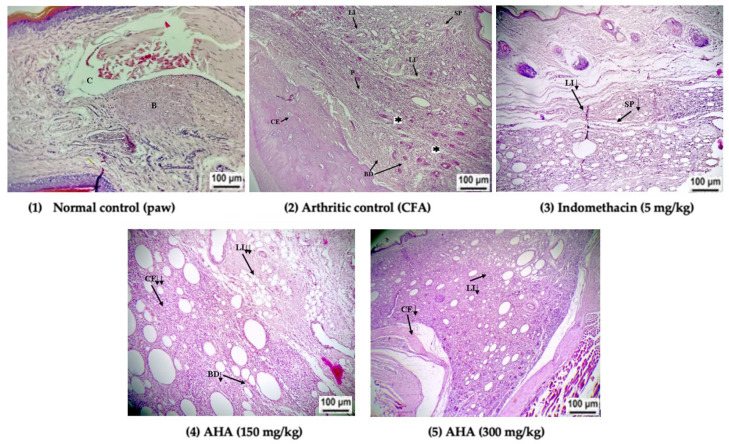
Microscopic evaluation (×10) of paw and liver tissues in CFA-induced rats. (**1**) Normal control showing normal bone (B) and cartilage (C) with no leukocyte infiltration; (**2**) Arthritic control: severe leukocyte infiltration (LI), synovial hyperplasia (SP), pannus formation (P), cartilage erosion (CE), and extensive bone destruction (BD); *: Areas of inflammatory cell infiltration. (**3**) Indomethacin (5 mg/kg)-treated group: marked reduction in leukocyte infiltration (LI↓) and synovial hyperplasia (SP↓), with preservation of cartilage and bone structures; (**4**) AHA-treated group (150 mg/kg): significant improvement with minimal leukocyte infiltration (LI↓↓), marked reduction in cartilage erosion (CE↓↓), and reduced bone destruction (BD↓) compared with the arthritic control. (**5**) AHA-treated group (300 mg/kg): partial improvement with decreased leukocyte infiltration (LI↓) and reduced cartilage erosion (CE↓) compared with the arthritic control.

Histopathological examination of paw tissue from normal control rats showed no evidence of inflammation, with an intact synovial lining and normal bone architecture Figure 7(1). In contrast, CFA-treated rats exhibited marked inflammatory cell infiltration composed mainly of monocytes, macrophages, lymphocytes, and polymorphonuclear cells. These changes were accompanied by bone destruction, chronic inflammatory alterations, disruption of the synovial lining, pannus formation invading the articular surface, and evident erosion of cartilage and subchondral bone Figure 7(2). Treatment with AHA extract (150 and 300 mg/kg) reduced these pathological alterations. Rats treated with 150 mg/kg showed notable protection, characterized by reduced inflammatory cell infiltration, a marked reduction in cartilage erosion, protection against bone destruction, and minimal pannus formation Figure 7(4). Although the 300 mg/kg dose also improved the histological features, mild inflammatory infiltration, reduced cartilage erosion, and partial tissue alterations were still observed Figure 7(5).

**Figure 8:**
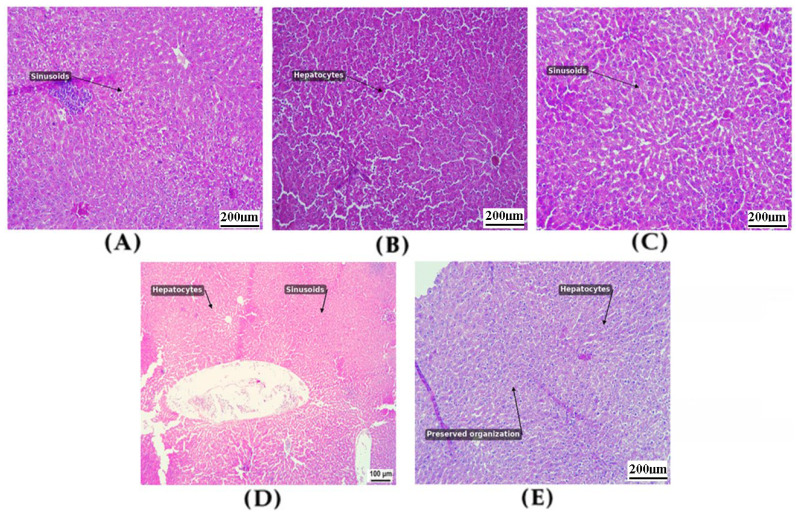
Microscopic evaluation of liver tissues in CFA-induced rats. (**A**) Liver tissue of the CFA-induced arthritic group (×20); (**B**) Normal liver tissue (×20); (**C**) Liver tissue from rats treated with AHA extract (150 mg/kg) (×20); (**D**) Liver tissue from rats treated with AHA extract (300 mg/kg) (×10); and (**E**) Liver tissue from rats treated with indomethacin (5 mg/kg) (×20).

Histopathological examination of liver sections from the CFA group Figure 8A revealed sinusoidal congestion associated with inflammatory cell infiltration compared to the normal group Figure 8B, which exhibited preserved hepatic cords and normal vascular architecture. In contrast, liver sections from rats treated with AHA extract (150 and 300 mg/kg) showed only mild vascular congestion with largely preserved hepatic architecture, suggesting a protective effect against CFA-induced hepatic injury Figure 8C,D.

## 3. Discussion

Natural antioxidants, particularly phenolic compounds, are widely recognized for their beneficial health effects. Polyphenols and flavonoids are major bioactive constituents responsible for the antioxidant potential of plant extracts, as they are capable of donating electrons or hydrogen atoms to neutralize free radicals [42,43]. In the present study, the total phenolic and flavonoid contents of the AHA extract were comparable to those reported by Benmarce et al. [19], who documented values of 19.60 ± 0.01 µg GAE/mg extract and 1.99 ± 0.02 µg QE/mg extract, respectively.

Because oxidative processes in biological systems are complex, a single method is not sufficient to fully evaluate antioxidant activity. Therefore, four complementary in vitro assays (DPPH, ABTS, CUPRAC, and *o*-phenanthroline) were performed to obtain a reliable and comprehensive assessment of the antioxidant capacity of the AHA extract. BHA and BHT were used as standard synthetic antioxidants due to their well-established non-enzymatic antioxidant properties. Each method measures antioxidant activity through a different mechanism. The DPPH assay evaluates the ability of antioxidants to neutralize a stable free radical in an organic medium [44]. The ABTS assay is more versatile, as it works in both aqueous and organic systems and detects both hydrophilic and lipophilic antioxidants [45]. The CUPRAC and *o*-phenanthroline methods measure the reducing power of the extract by assessing its ability to convert metal ions (Cu^2+^ or Fe^3+^) into their reduced forms [46,47]. Together, these assays allow a broader assessment of radical-scavenging and electron-donating capacity. Antioxidant activity was expressed as IC_50_ values for DPPH and ABTS, and as A_0.5_ values for CUPRAC and *o*-phenanthroline assays. Lower IC_50_ and A_0.5_ values indicate higher antioxidant activity. The extract showed variable activity across assays, with the strongest effect observed in the *o*-phenanthroline method and the weakest in the DPPH assay. Overall, the antioxidant effectiveness followed the order: phenanthroline > ABTS > CUPRAC > DPPH. As expected, the synthetic standards BHA and BHT exhibited much stronger activity than the extract. The antioxidant performance of the extract is consistent with its phenolic and flavonoid content. Phenolic compounds can donate hydrogen atoms or electrons to neutralize free radicals due to the presence of hydroxyl groups attached to aromatic rings [48]. Therefore, extracts containing higher levels of phenolics and flavonoids generally show stronger activity in radical-scavenging and reducing-power assays. The results obtained in this study suggest that the extract’s antioxidant capacity is primarily attributable to its polyphenolic content.

The aqueous extract of *A. halimus* exerts a differential effect on cancerous and non-tumorigenic cells. A moderate, concentration-dependent reduction in viability was observed in HeLa cells, particularly at higher concentrations, indicating a cytotoxic effect, although limited in magnitude.

In contrast, HaCaT cells were not adversely affected by the treatment. Instead, cell viability was maintained or even increased at higher concentrations, suggesting a possible cytoprotective or stimulatory effect. This selective response may be related to differences in cellular metabolism and redox status between cancer and normal cells, as previously reported for plant-derived extracts.

A limitation of the present study is the use of 5% DMSO as a positive control in the cytotoxicity assay. Although DMSO is commonly employed as a cytotoxic control because of its membrane-disrupting properties at high concentrations, its cytotoxic effect is strongly influenced by the intrinsic susceptibility of different cell lines. In the present study, 5% DMSO induced almost complete cell death in HeLa cells, whereas HaCaT cells retained partial viability, indicating a cell line-dependent response. Therefore, while DMSO served as a reference for maximal cytotoxicity, it is not an ideal positive control for assessing selective cytotoxic effects. Future studies should include established chemotherapeutic agents with well-characterized mechanisms of action as positive controls to further validate the selective cytotoxic activity of AHA.

However, the increase in HaCaT viability should be interpreted cautiously, as it may reflect enhanced metabolic activity rather than a true proliferative effect. Further studies are needed to clarify the underlying mechanisms and to identify the bioactive compounds responsible for the observed effects.

Overall, these findings suggest that the aqueous extract of *A. halimus* exhibits low toxicity toward normal cells and moderate activity against cancer cells, supporting its potential as a source of biologically active compounds.

AHA is capable of inducing DNA damage at higher concentrations, as evidenced by the significant increase in Comet assay parameters. In particular, the elevation of % tail DNA, tail moment, and olive tail moment at 50 and 100 µg/mL indicates the occurrence of DNA strand breaks and confirms a concentration-dependent genotoxic effect.

The absence of significant effects at the lowest concentration (6 µg/mL) suggests the existence of a threshold below which the extract does not compromise DNA integrity. This concentration-dependent behavior is commonly reported for plant-derived extracts and may reflect the balance between protective and damaging cellular responses depending on the dose [49].

The observed genotoxicity may be associated with the presence of bioactive compounds capable of altering the cellular redox balance or interacting with DNA, leading to strand breaks [50]. Moreover, under cell culture conditions, certain phenolic compounds may undergo autoxidation, generating hydrogen peroxide and other reactive oxygen species that can exert pro-oxidant effects and contribute to DNA damage, particularly at higher concentrations [51,52]. In cancer-related models, such effects can be considered relevant, as DNA damage may contribute to the activation of apoptotic pathways and the inhibition of the cell cycle [53].

The modulation of IL-1β gene expression observed in this study provides important mechanistic insight into the anti-inflammatory activity of the *Atriplex halimus* aqueous extract (AHA). IL-1β is a key pro-inflammatory cytokine involved in the pathogenesis of rheumatoid arthritis, where it promotes synovial inflammation, cartilage degradation, and bone erosion through activation of NF-κB-dependent inflammatory pathways [54,55]. To investigate the anti-inflammatory potential of AHA, we employed a zymosan-induced inflammatory model. Zymosan, a β-glucan-rich polysaccharide derived from the cell wall of *Saccharomyces cerevisiae*, is widely used as an experimental inflammatory stimulus because it activates pattern recognition receptors, particularly Toll-like receptor 2 (TLR2) and dectin-1 [56]. Activation of these pathways triggers NF-κB signaling and promotes the production of pro-inflammatory mediators, including IL-1β, TNF-α, and IL-6. Therefore, this model provides a suitable platform for evaluating the ability of natural compounds to modulate innate immune and inflammatory responses. In our model, zymosan stimulation markedly increased IL-1β mRNA expression, confirming activation of TLR2-mediated inflammatory signaling and inflammasome-related responses [57].

Pre-treatment with AHA significantly reduced zymosan-induced IL-1β expression, demonstrating its ability to attenuate inflammatory transcriptional activation. This effect is consistent with the phytochemical composition of the extract, which is rich in methoxylated flavonol glycosides, particularly isorhamnetin- and syringetin-derived compounds, as well as caffeic acid derivatives. These polyphenols are widely reported to suppress NF-κB activation, inhibit IL-1β production, and modulate NLRP3 inflammasome signaling [58,59].

The inhibitory effect on IL-1β expression also correlates with the strong antioxidant activity of AHA observed both in vitro and in vivo. Since oxidative stress is a major trigger of NF-κB and inflammasome activation, the reduction in NO and MDA levels together with the restoration of GSH and CAT activity likely contributed to the downregulation of inflammatory mediators [60]. Hydroxycinnamic acids and flavonoids identified in the extract are known to act as ROS scavengers and regulators of redox-sensitive inflammatory pathways [61].

Overall, these findings suggest that the anti-inflammatory and anti-arthritic effects of AHA are at least partially mediated through the modulation of IL-1β-related signaling pathways, likely driven by the synergistic action of its phenolic constituents.

Protein denaturation is a process in which native proteins lose their secondary and tertiary structures due to the disruption of hydrogen, hydrophobic, and disulfide bonds [62]. As a result, proteins lose their biological function. In inflammatory conditions, denatured proteins may act as autoantigens, triggering immune responses. Denatured albumin, in particular, can induce type III hypersensitivity reactions, serum sickness, and glomerulonephritis, all of which are associated with inflammatory and autoimmune mechanisms [63]. In rheumatoid arthritis, structural modification of endogenous proteins (such as citrullination) can generate neo-antigens that are recognized by the immune system, promoting autoreactive T and B cell responses and contributing to chronic inflammation in the joints [64]. Therefore, inhibition of protein denaturation is considered an important mechanism in the prevention and management of arthritis. The observed anti-denaturation activity may be attributed to the phenolic and flavonoid compounds present in the AHA extract. These compounds are known to interact with proteins and protect them from structural alteration through antioxidant and anti-inflammatory mechanisms [61].

*Atriplex halimus* is widely used in folk medicine for the management of rheumatic pain [17]. In the present study, the anti-arthritic potential of AHA was evaluated using a CFA-induced rat model, which causes bone destruction by increasing bone resorption and reducing bone formation [65], providing a clinically relevant experimental framework.

Adjuvant-induced arthritis in rats causes bone destruction by increasing bone resorption and reducing bone formation [66], providing a clinically relevant framework for evaluating novel anti-arthritic agents. AHA extract significantly inhibited paw edema and ankle joint inflammation during both the acute and chronic stages of arthritis. Notably, the 150 mg/kg dose demonstrated superior anti-inflammatory effects compared to the 300 mg/kg dose, suggesting an inverse dose–response relationship.

CFA-induced arthritic rats exhibited reduced weight gain consistent with rheumatoid cachexia, a condition driven by cytokine-mediated hypermetabolism and activation of the ubiquitin–proteasome pathway [67]. AHA treatment attenuated this weight loss during the second week of the experiment, likely by downregulating pro-inflammatory cytokines such as TNF-α and IL-1β, central mediators of muscle wasting in RA [68]. This systemic protective effect suggests that AHA’s anti-inflammatory action extends beyond localized joint tissue. Similarly, spleen weight was reduced in AHA-treated animals relative to arthritic controls, indicating attenuation of systemic immune hyperactivity, consistent with reports showing that flavonoid-rich extracts modulate B- and T-cell responses and reduce lymphoid organ hypertrophy [69,70].

Hematological analysis revealed the characteristic profile of CFA-induced arthritis, including reduced RBC count and hemoglobin levels reflecting anemia of chronic inflammation [71,72], alongside elevated WBC and platelet count indicative of sustained immune activation and inflammatory cascade amplification [73,74,75]. AHA extract treatment corrected these disturbances by restoring RBC and hemoglobin levels while reducing WBC and platelet counts toward normal values, demonstrating a broad hematological normalization effect consistent with its anti-inflammatory and immunomodulatory properties.

Biochemical assessments of serum AST, ALT, urea, and creatinine were performed to evaluate the hepatic and renal safety of the extract [76]. Elevated AST and ALT reflect hepatocellular damage, while increased urea and creatinine indicate impaired renal filtration capacity under pathological conditions [77,78]. AHA treatment maintained all these parameters closer to normal values at both tested doses, confirming a hepatoprotective and nephroprotective profile and indicating that the extract is both effective and safe within the tested dose range.

CFA-induced arthritis produced marked oxidative stress in the serum, liver, and spleen, as evidenced by significantly elevated MDA and NO levels alongside depleted GSH content and catalase activity compared to normal controls, confirming the central role of oxidative damage in arthritis pathophysiology. AHA extract treatment at both doses effectively reduced MDA and NO levels while restoring GSH and catalase activity, with effects comparable to indomethacin. The lower dose (150 mg/kg) again showed slightly superior normalization of oxidative biomarkers, reinforcing the notion of a non-linear or biphasic dose–response relationship rather than a simple dose-independent phenomenon. This behavior may be partially attributed to antinutritional factors such as phytates and tannins, which reduce phytochemical bioavailability at higher concentrations [79], or to gastric oversaturation leading to precipitation and reduced absorption of active constituents [80]. More importantly, this non-linear response strongly suggests a paradoxical pro-oxidant activity of polyphenolic compounds at elevated concentrations [81]. At a high dose (300 mg/kg), abundant flavonoids and phenolic acids can undergo autoxidation pathways, particularly in the presence of trace transition metals. This process generates phenoxyl radicals and reactive oxygen species (ROS) instead of neutralizing them, thereby accelerating the consumption of endogenous antioxidants like GSH and paradoxically exacerbating rather than attenuating oxidative stress [82]. High-dose phytochemicals may also interfere with hepatic cytochrome P450 enzymes, potentially disrupting GSH synthesis pathways and consequently diminishing the extract’s overall antioxidant and anti-inflammatory efficacy [83].

These antioxidant effects are consistent with the extract’s rich polyphenol and flavonoid content and align with previous studies reporting AHA-mediated protection against oxidative damage induced by toxic agents such as benzene, sodium benzoate, and carbon tetrachloride [84,85].

Finally, histopathological examination confirmed the biochemical and functional findings. Arthritic control rats displayed marked synovial hyperplasia, inflammatory cell infiltration, and structural joint alterations, faithfully reproducing key features of human RA [86], along with hepatic sinusoidal congestion and focal inflammatory infiltrates reflecting systemic inflammatory involvement [87]. In contrast, AHA-treated rats showed significantly reduced inflammatory cell infiltration and improved tissue architecture preservation in both paw and liver sections, providing morphological confirmation of the extract’s anti-inflammatory and cytoprotective efficacy in experimental arthritis.

From a translational perspective, the anti-arthritic effects of AHA extract may be partly mediated through modulation of the NLRP3 inflammasome and mitogen-activated protein kinase (MAPK) signaling pathways. The NLRP3 inflammasome regulates the maturation of IL-1β, a pivotal cytokine involved in synovial inflammation, cartilage degradation, and bone erosion in rheumatoid arthritis (RA). Polyphenols have been reported to suppress NLRP3 activation by reducing mitochondrial reactive oxygen species (ROS) production and inhibiting caspase-1 activation, thereby limiting IL-1β maturation and downstream inflammatory responses [88]. Likewise, the MAPK signaling cascades, including extracellular signal-regulated kinase (ERK1/2), p38, and c-Jun *N*-terminal kinase (JNK), are key regulators of pro-inflammatory cytokine production, synovial fibroblast proliferation, and pannus formation. Several flavonoids have been shown to inhibit MAPK phosphorylation, resulting in reduced NF-κB activation and attenuation of inflammatory signaling [89]. Although these molecular pathways were not directly investigated in the present study, the marked reduction in synovial hyperplasia, inflammatory cell infiltration, and tissue destruction observed in AHA-treated rats is consistent with these proposed mechanisms.

To facilitate the clinical translation of the present findings, the effective animal doses were converted to human-equivalent doses (HEDs) using the body surface area normalization method described by Nair and Jacob [90]. Applying a rat Km factor of 7 (250–300 g rats) and a human Km factor of 37, the 150 mg/kg dose corresponds to an HED of approximately 28.4 mg/kg (approximately 1.7 g/day for a 60 kg adult), whereas the 300 mg/kg dose corresponds to approximately 56.8 mg/kg (approximately 3.4 g/day). Following the recommended safety factor of 10 for first-in-human studies, the maximum recommended starting dose derived from the more effective 150 mg/kg dose would be approximately 2.84 mg/kg (approximately 170 mg/day). It should be noted, however, that HED calculations provide only an initial estimate for dose translation and do not account for interspecies differences in pharmacokinetics, metabolism, or oral bioavailability.

The relationship between the effective in vitro concentrations (50–100 μg/mL) and the in vivo doses (150–300 mg/kg) cannot be directly established because systemic exposure depends on multiple pharmacokinetic factors, including gastrointestinal absorption, first-pass metabolism, plasma protein binding, tissue distribution, and elimination. Since pharmacokinetic data for AHA extract are currently unavailable, the plasma concentrations achieved following oral administration remain unknown. Therefore, although both the in vitro and in vivo experiments demonstrated significant biological activity, a quantitative exposure-response relationship cannot yet be determined.

## 4. Materials and Methods

### 4.1. Plant Collection and Authentication

*Atriplex halimus* L. was collected in May 2023 from Setif, northern Algeria (35.6815° N, 5.1919° E). The plant material was botanically identified and authenticated by Dr. Wafa Nouioua (Department of Plant Ecology and Biology, Faculty of Natural and Life Sciences, Ferhat Abbas University Setif 1, Algeria), following the taxonomic keys of Quézel and Santa (1962–1963). A voucher specimen (UFS-2024-03) was deposited at the Laboratory of Phytotherapy Applied to Chronic Diseases.

### 4.2. Chemicals

All reagents and chemicals used in this study were of analytical grade and acquired from Sigma Aldrich potassium persulfate (K_2_S_2_O_8_), ABTS, ethanol, DPPH, butylated hydroxyanisole (BHA), butylated hydroxytoluene (BHT), ammonium acetate (ACNH_4_), copper(II) chloride (CuCl_2_), neocuprione, *o*-phenanthroline, ferric chloride (FeCl_3_), methanol (MeOH), Folin–Ciocalteu reagent, vanillin, aluminum chloride (AlCl_3_), and Complete Freund’s Adjuvant (CFA), 5,5′-Dithiobis(2-nitrobenzoic acid) (DTNB), Thiobarbituric acid (TBA), Trichloroacetic acid (TCA), n-Butanol, Hydrogen peroxide (H_2_O_2_), Sulfanilamide, *N*-(1-naphthyl)ethylenediamine dihydrochloride (NEDD), Phosphoric acid.

### 4.3. Animals

Adult male Wistar rats weighing 250–300 g were obtained from the breeding facilities of the Pasteur Institute (Algiers, Algeria) and housed in the experimental animal facility of the Biochemistry Department, Faculty of Natural and Life Sciences, Setif, Algeria. The animals were maintained under standard laboratory conditions conducive to normal growth and development, with free access to food supplied by the National Office for Livestock Feed (ONAB) and drinking water. All experimental procedures were conducted in accordance with the ethical guidelines of the Algerian Association of Experimental Animal Sciences (Law No. 88-08/1988) and complied with the European Union Directive on the protection of animals used for scientific purposes (2010/63/EU), ensuring adherence to animal welfare and ethical standards.

### 4.4. Preparation of Aqueous Extracts

Dried plant material was finely powdered using an electric grinder. One hundred grams (100 g) of the powdered plant material was macerated in 1 L of distilled water (solid-to-solvent ratio 1:10, *w*/*v*) as previously described by [91] for 48 h at room temperature with occasional stirring. The mixture was then filtered through Whatman No. 1 filter paper to remove plant debris. The filtrate was concentrated under reduced pressure using a rotary evaporator at 40–45 °C and subsequently dried to obtain the crude aqueous extract. The dried extract was weighed to determine the extraction yield and stored at 4 °C until further use.

### 4.5. Determination of the Total Phenolic Contents (TPC)

The extract of *A. halimus* was determined for total phenolic contents by the Folin–Ciocalteu assay [92] using a calibration curve of standard gallic acid. A total of 100 µL of the sample at 1 mg/mL was placed in a test tube, then 400 µL of sodium carbonate solution (7.5%) was added, followed by the addition of 500 µL of Folin–Ciocalteu reagent solution. After 2 h of incubation at room temperature, the absorbance was measured at 765 nm against the blank. The results are expressed as ug gallic acid equivalent per mg dry extract.

### 4.6. Determination of the Total Flavonoid Content (TFC)

The extract of *A. halimus* was determined for total flavonoid contents by the aluminum chloride assay [93] using a calibration curve of standard quercetin. A total of 1 mL of extract solution was mixed with 1 mL of methanolic AlCl_3_. The absorbance was measured 10 min later at 430 nm against the blank. The results are expressed as ug of quercetin equivalent per mg of dry extract.

### 4.7. Condensed Tannin Content (CTC)

The condensed tannins tenor has been performed using a modifying vanillin procedure [94]. A total of 250 μL of the samples was combined with 375 μL of vanillin reagent (4%). Later, 187.5 μL of HCl was added. The resulting blend has been incubated for twenty minutes. The spectrophotometric measurement was conducted at 500 nm. The data obtained were reported in ug of catechin equivalent per mg of dry extract (μg CE/mg DE).

### 4.8. LC–HRMS/MS Analysis

LC–HRMS and LC–HRMS/MS analyses were performed on a Thermo Scientific Orbitrap Exploris 120 high-resolution mass spectrometer (Thermo Fisher Scientific, Waltham, MA, USA) equipped with a heated electrospray ionization (HESI) source and coupled to a Thermo Dionex Ultimate 3000 UHPLC system. Chromatographic separation was carried out on a Thermo scientific hypersil (150 mm × 3 mm, 3 μm), maintained at room temperature. The mobile phases were water with 0.1% formic acid (A) and acetonitrile (B), delivered at 0.450 mL min^−1^. The gradient was as follows: 5% B (0–2 min), 5–50% B (2–10 min), isocratic at 50% B (10–12 min), 50–95% B (12–18 min), isocratic at 95% B (18–20 min) and 95–5% B (20.1–24 min). The injection volume was 5 μL. Mass spectra were acquired in negative ion modes. HESI source parameters were spray voltage 3.5 kV (positive) and 2.3 kV (negative), capillary temperature 320 °C, sheath gas 50 arb, and auxiliary gas 10 arb. Full MS scans were acquired from *m*/*z* 110 to 1500. Data-dependent acquisition (DDA) was used, selecting the top 4 most intense precursor ions from each full scan for MS/MS. Fragmentation was performed using higher-energy collisional dissociation (HCD) with a normalized collision energy (NCE) of 30%. Raw data were processed using Thermo Scientific FreeStyle software (version 1.8).

### 4.9. In Vitro Antioxidant Activity

#### 4.9.1. DPPH Free Radical Scavenging Assay

The free radical scavenging activity of the plant extract was evaluated spectrophotometrically using the 2,2-diphenyl-1-picrylhydrazyl (DPPH) assay according to the method described by Blois (1958) [95], with slight modifications. In each well of a 96-well microplate, 160 µL of DPPH solution was mixed with 40 µL of the plant extract at different concentrations. The mixture was incubated at room temperature in the dark for 30 min. The absorbance was then measured at 517 nm using a microplate reader against a blank containing methanol.

#### 4.9.2. ABTS Discoloration Assay

The ABTS 2,2-azinobis (3-ethylbenzothioazolin-6-sulfonic acid) radical scavenging activity was evaluated according to the method of Re et al. [45], with slight modifications. The ABTS radical cation was generated by mixing 7 mM ABTS solution with 2.45 mM potassium persulfate, and the mixture was allowed to react for 12–16 h at room temperature in the dark. Before use, the resulting ABTS•^+^ solution was diluted with distilled water to obtain an absorbance of 0.700 ± 0.020 at 734 nm. For the assay, 160 µL of the diluted ABTS solution was mixed with 40 µL of the extract solution, and the reaction mixture was incubated for 10 min at room temperature in the dark. The absorbance was then measured at 734 nm using a microplate reader. BHA was used as a positive control.

#### 4.9.3. Curic Reducing Antioxidant Capacity Assay

The cupric reducing antioxidant capacity (CUPRAC) of the extract was determined according to the method described by Apak et al. [46] with slight modifications. Briefly, in each well, 50 µL of 10 mM copper (II) chloride solution, 50 µL of 7.5 mM neocuproine solution, and 60 µL of ammonium acetate buffer (1 M, pH 7.0) were mixed. Subsequently, 40 µL of the plant extract at different concentrations was added to initiate the reaction. The mixture was incubated at room temperature for 60 min. After incubation, the absorbance was measured at 450 nm against a reagent blank using a microplate reader. The antioxidant capacity was expressed as A_0.5_ (µg/mL), defined as the concentration of extract required to produce an absorbance of 0.50 at 450 nm.

#### 4.9.4. *o*-Phenanthroline Free Radical Reducing Activity

The *o*-phenanthroline free radical reducing activity was evaluated following the method of Szydłowska-Czerniak et al. [47]. Briefly, a reaction mixture containing 30 µL of *o*-phenanthroline solution (0.5%), 50 µL of FeCl_3_ (0.2%), and 110 µL of methanol was prepared and combined with 10 µL of plant extract at various concentrations. The mixture was incubated at 30 °C for 20 min, after which the absorbance was recorded at 510 nm. The antioxidant activity was expressed as the percentage of inhibition. The antioxidant capacity was expressed as A_0.5_ (µg/mL).

### 4.10. In Vitro Anti-Inflammatory Activity

#### Inhibition of Albumin Denaturation

The anti-inflammatory effect of AHA was evaluated using the heat-induced bovine serum albumin (BSA) denaturation assay, according to the method described by [96]. The tested extract or diclofenac sodium (standard) at different concentrations was mixed with 0.2% bovine serum albumin (BSA) prepared in Tris-buffered saline (pH 6.8). The mixtures were incubated at 37 °C for 20 min and then heated at 70 °C for 10 min, followed by cooling. The absorbance of the test and control solutions was measured at 660 nm. The percentage inhibition of protein denaturation was calculated by using the following formula:

% Inhibition = Abs control − Abs test/Abs control × 100

### 4.11. In Vitro Cytotoxicity Activity

The cytotoxic activity was evaluated using the Cell Counting Kit-8 (CCK-8; Dojindo Molecular Technologies, Kumamoto, Japan), following the manufacturer’s instructions.

HeLa cells (human cervical cancer) were purchased from ATCC (CCL-2), while HaCaT (immortalized human keratinocyte) cells were purchased from CLS Cell Lines Service, Germany. Both cell lines were seeded in 96-well plates at a density of 3 × 10^3^ cells/well in 100 μL of complete Dulbecco’s Modified Eagle Medium (DMEM) and incubated at 37 °C in a 5% CO_2_ humidified atmosphere for 24 h to allow cell adherence.

Cells were then treated with increasing concentrations of AHA (100, 50, 25, 12.5, 6.25, 3.125, 1.56 µg/mL), diluted in DMSO with the final DMSO concentration 0.1% and incubated for 24 h. Untreated cells (DMEM + 0.1% DMSO) were used as negative controls. Wells containing medium and CCK-8 reagent without cells were included as blanks.

After treatment, 10 μL of CCK-8 reagent was added to each well, and the plates were incubated for 2 h at 37 °C. The absorbance was measured at 450 nm using a microplate reader (Bio-Rad, Thermo Fisher, Hercules, CA, USA).

### 4.12. Genotoxicity Assessment by Comet Assay

DNA damage induced by the AHA extract was assessed in HeLa cells using the comet assay, following the protocol by Singh et al. [48], with minor modifications according to Dentato et al. [97], and 4 × 10^5^ HeLa cells were seeded in 60 mm dishes. After 24 h, cells were treated with various concentrations of the extract (6, 50 and 100 µg/mL) for 24 h, harvested, and embedded in 1% low-melting agarose on microscope slides precoated with 1% normal-melting agarose.

After lysis (2.5 M NaCl, 100 mM EDTA, 10 mM Tris, 1% Triton X-100, pH 10) for 1 h at 4 °C, slides were placed in electrophoresis buffer (300 mM NaOH, 1 mM EDTA, pH > 13) for 20 min to allow DNA unwinding. Electrophoresis was performed at 25 V and 300 mA for 20 min. Slides were neutralized with Tris buffer (0.4 M, pH 7.5) and stained with ethidium bromide (5 µg/mL) (Sigma-Aldrich, Darmstadt, Germany).

A fluorescence microscope was used to examine the slides, analyzing a minimum of 50 randomly selected nuclei from each slide and avoiding overlapping figures. A computerized image-analysis system (ImageJ BioRad software, plugin OpenComet, Version 2.9.0) was employed. Twenty-five nuclei were scored per slide, three slides were evaluated per treatment, and each treatment was repeated at least twice. From the repeated experiments, DNA in the tail, tail moment, and olive moment from each slide were calculated.

### 4.13. Analysis of IL-1β Gene Expression

Since oxidative stress and inflammation are tightly interconnected processes, and IL-1β represents a key mediator linking these pathways, we assessed IL-1β gene expression to further explore the extract’s potential modulatory effects on the inflammatory response.

To evaluate whether pretreatment with the extract regulates IL-1β gene expression, cells were pre-incubated with the extract for 24 h, followed by stimulation with zymosan for 24 h. IL-1β mRNA levels were quantified by RT-qPCR.

HaCaT cells were seeded at a density of 2.5 × 10^5^ cells per 60 mm culture dish and allowed to adhere overnight. Cells were then pretreated for 24 h with 50 μg/mL of AHA extract, vehicle control (0.1% DMSO), or the positive control quercetin (1.5 μM) (Q0125, Sigma-Aldrich, Darmstadt, Germany).

After 24 h, cells were stimulated with 75 μg/mL zymosan (Z4250, Sigma-Aldrich, Darmstadt, Germany)—for inducing an inflammatory response- for an additional 24 h. Two additional control conditions were included: cells treated with 0.1% DMSO alone and cells pretreated with the extract followed by 0.1% DMSO (without zymosan), both incubated for 24 h.

Total RNA was extracted using the VWR Total RNA Purification Kit according to the manufacturer’s instructions. RNA concentration and purity were determined spectrophotometrically. For reverse transcription, 1 μg of total RNA was converted into complementary DNA (cDNA) using the Vazyme HiScript Reverse Transcription Master Mix following the supplier’s protocol. Quantitative real-time PCR (RT-qPCR) was performed using Vazyme 2× Taq Pro Universal SYBR qPCR Master Mix on an Applied Biosystems StepOnePlus™ real-time PCR system. Primers for human IL-1β (Fw: 5′-CCACAGACCTTCCAGGAGAATG-3′; Rv: 5′-GTGCAGTTCAGTGATCGTACAGG-3′) and the reference gene GAPDH (Fw: TTGCCATCAATGACCCCTTCA Rv: CGCCCCACTTGATTTTGGA) were purchased from Euroclone. The thermal cycling conditions were as follows: initial denaturation at 95 °C for 30 s, followed by 40 cycles of 95 °C for 5 s and 60 °C for 30 s. Relative gene expression was calculated using the 2^−ΔΔCt^ method, with GAPDH as the endogenous normalizer. All reactions were performed in technical triplicate.

### 4.14. In Vivo Antiarthritic Activity

#### 4.14.1. Complete Freund’s Adjuvant (CFA)-Induced Arthritis

Five groups of seven albino male rats, each weighing between 250 and 300 g, were established. Group 1 served as the normal control group, Group 2 received vehicle (0.01% Tween 80) for arthritis, and Group 3 was treated with indomethacin (5 mg/kg p.o.). Groups 4 and 5 received the aqueous extracts at concentrations of 150 and 300 mg/kg, respectively. They were administered once daily by oral gavage. Arthritis was induced by intradermal injection of Freund’s complete adjuvant (CFA). The adjuvant contained 10 mg of heat-killed *Mycobacterium tuberculosis* in 1 mL of paraffin oil. On the initial day, 0.1 mL of complete Freund’s adjuvant was injected into the subplantar region of the right hind paw of each rat. Testing commenced on the same day and continued for 21 days. On the 21st day, the animals were anesthetized with ether. Blood was collected from the retro-orbital puncture, and an overdose of ether subsequently sacrificed the animals [98].

#### 4.14.2. Measurement of Body Weight

Body weight was measured on the initial day (day 0) immediately before the CFA injection and subsequently on days 1, 4, 8, 12, 16, and 20.

#### 4.14.3. Measurement of Paw Diameter and Ankle Joint

A digital Vernier caliper was used for measurement of paw and joint diameter on day 0 before CFA injections and subsequently on days 1, 4, 8, 12, 16, and 20.

#### 4.14.4. Measurement of Arthritis Score

The development and severity of CFA-induced arthritis were evaluated using a visual scoring system of clinical signs and symptoms on a scale of 0–4 per limb, as described by Van Eden et al. [99]. The scoring criteria were 0—no change; 1—slight swelling and erythema of the limb; 2—mild swelling and erythema; 3—gross swelling and erythema; and 4—gross deformity and inability of the limb. Measurements were initiated on day 1 after CFA injection and subsequently on days 4, 8, 12, 16, and 20.

#### 4.14.5. Measurement of Spleen Weight

All the rats were sacrificed after the 21st day following anesthesia with ether. The spleen was removed from each rat, cleaned, and weighed.

#### 4.14.6. Measurement of Hematological Parameters

Before termination of the experiment on day 21, blood samples were collected by the retro-orbital route under ether anesthesia, using glass capillary tubes into Ethylene Diamine Tetra-acetic Acid (EDTA)-coated sample bottles for full blood count determination.

#### 4.14.7. Measurement of Biochemical Parameters

Before termination of the experiment on day 21, serum samples were analyzed for urea, creatinine, aspartate aminotransferase (AST), and alanine aminotransferase (ALT).

#### 4.14.8. Measurement of Serum, Liver, and Spleen GSH, NO, Cat, and MDA

Blood samples were collected via retro-orbital puncture, and liver and spleen tissues were harvested to prepare homogenates. Levels of GSH, NO, catalase (Cat), and MDA were determined in serum as well as in liver and spleen homogenates using the methods described by [100,101,102,103], respectively.

#### 4.14.9. Preparation of Homogenates

Portions of liver and spleen from each rat were homogenized in 50 mM Tris-HCl buffer (pH 7.4) using a homogenizer. Each homogenate was then centrifuged at 4000× *g* at 4 °C for 15 min (Sigma 3–30K, Germany). The resulting supernatants were collected and stored at −20 °C for further analyses.

#### 4.14.10. Histopathological Examination of the Paw Tissue and Liver

On the 21st day, the rats were euthanized. All samples were then transported to the Pathology Laboratory of the CAC-Setif, Algeria, for further analysis. The arthritic paw, liver tissues were removed and preserved in buffered formalin (10%) for 14 days. The paw tissue was decalcified in 5% formic acid, fixed in paraffin, and sectioned at 5 μm. The sections were stained with hematoxylin-eosin solution and analyzed under a light microscope [104].

### 4.15. Statistical Analysis

The results of the in vitro tests were expressed as mean ± standard deviation in triplicate. The results in vivo were presented as mean ± SEM and statistically analyzed using GraphPad Prism version 10. One- and two-way ANOVA followed by the Dunnett’s test were applied as appropriate. Two-way ANOVA was used to analyze the in vitro antiarthritic activity, paw diameter, body weight, and hematological and biochemical parameters in the CFA-induced arthritis model. One-way ANOVA was used to evaluate oxidative stress biomarkers, cytotoxicity, genotoxicity effects and gene expression.

## 5. Conclusions and Future Perspective

Many plants contain phytochemicals that play important roles in human life and provide beneficial effects. In the present study, aqueous extracts of *Atriplex halimus* leaves demonstrated notable antioxidant activity. The extract inhibited protein denaturation and exhibited selective cytotoxic activity against HeLa cells while showing no significant toxicity toward normal HaCaT cells. In addition, genotoxicity assessment indicated DNA damage at higher concentrations, suggesting a concentration-dependent effect. The extract also significantly reduced paw swelling, arthritic scores, and joint tissue damage in CFA-induced arthritic rats. It improved hematological, biochemical, and antioxidant parameters, indicating protection against systemic complications such as cachexia and oxidative stress. These effects are likely associated with the presence of phenolic compounds, flavonoids, and tannins, which contribute to the modulation of inflammatory mediators, membrane stabilization, and attenuation of oxidative damage. Overall, *A. halimus* extract shows promise as a natural therapeutic agent for rheumatoid arthritis and as a potential source of selective anticancer compounds. Future studies should focus on the isolation of its active phytochemicals to elucidate their structure–activity relationships and underlying molecular mechanisms while further evaluating their safety profile.

## Figures and Tables

**Table 3:** In vitro antioxidant activity of the aqueous extract of AHA determined by DPPH, ABTS radical scavenging, CUPRAC, and phenanthroline assays in comparison with BHA and BHT. Data are presented as mean ± SD (n = 3); statistical analysis was performed using one-way ANOVA (**** *p* < 0.0001).

IC_50_ (µg/mL)		A_0.5_ (µg/mL)		
Samples	DPPH	ABTS	CUPRAC	*o*-Phenanthroline
AHA	697.77 ± 2.08 ****	411.28 ± 0.97 ****	665.22 ± 2.32 ****	136.68 ± 1.55 ****
BHT	12.99 ± 0.41	1.29 ± 0.30	9.62 ± 0.87	-
BHA	6.14 ± 0.41	1.82 ± 0.10	3.64 ± 0.19	2.24 ± 0.17

**Table 4:** Effect of AHA extract on hematological parameters in an arthritic rat model. Values are expressed as mean ± SEM (n = 7). Statistical analysis was performed using two-way ANOVA followed by Dunnett’s post hoc test. **** *p* < 0.0001; ^ns^ indicates non-significant differences.

Group	RBC (10^12^/L)	PLT (10^9^/L)	WBC (10^9^/L)	Hb (g/dL)
Arthritic control	7.53 ± 0.25	993 ± 19.65	15.46 ± 0.93	14.66 ± 0.93
Normal control	8.85 ± 0.22 ^ns^	747 ± 12.62 ****	13.68 ± 1.27 ^ns^	15.93 ± 0.22 ^ns^
Indomethacin	7.84 ± 0.07 ^ns^	783.33 ± 16.66 ****	13.53 ± 0.43 ^ns^	15.35 ± 0.41 ^ns^
AHA 300 mg/kg	7.71 ± 0.10 ^ns^	898.66 ± 6.63 ****	14.66 ± 0.56 ^ns^	15.36 ± 0.16 ^ns^
AHA 150 mg/kg	7.91 ± 0.22 ^ns^	867.66 ± 10.10 ****	13.72 ± 0.70 ^ns^	15.18 ± 0.10 ^ns^

**Table 5:** Effect of AHA extract on biochemical and hematological parameters in an arthritic rat model. Values are expressed as mean ± SEM (n = 7). Statistical analysis was performed using two-way ANOVA followed by Dunnett’s post hoc test. * *p* < 0.05, ** *p* < 0.01, *** *p* < 0.001; ^ns^ indicates non-significant differences.

Group	AST (U/L)	ALT (U/L)	Urea (g/L)	Creat (mg/dL)
Arthritic control	87.41 ± 1.21	58.23 ± 1.09	0.38 ± 0.04	8.42 ± 0.24
Normal control	65.01 ± 5.28 **	44.99 ± 1.60 **	0.31 ± 0.01 ^ns^	4.37 ± 1.40 **
Indomethacin	83.25 ± 3.26 ^ns^	44.01 ± 1.18 **	0.30 ± 0.03 ^ns^	3.46 ± 0.49 ***
AHA 300 mg/kg	72.23 ± 0.98 ^ns^	44.39 ± 4.20 **	0.35 ± 0.01 ^ns^	4.07 ± 0.01 ***
AHA 150 mg/kg	69.86 ± 7.81 *	41.52 ± 3.10 ***	0.34 ± 0.03 ^ns^	3.78 ± 0.59 ***

**Table 6:** Serum Levels of Nitric Oxide (NO), Reduced Glutathione (GSH), Malondialdehyde (MDA), and Catalase (CAT). Values are expressed as mean ± SEM (n = 7). Statistical analysis was performed using one-way. * *p* < 0.05, ** *p* < 0.01, **** *p* < 0.0001; ^ns^ indicates non-significant differences.

Group	GSH (nmole/mg Protein)	MDA (nmole/g Tissue)	NO (µM)	Cat (U/mg of Protein)
Arthritis control	4.51 ± 0.54	79.23 ± 1.17	4.04 ± 1.88	3.73 ± 0.35
Normal control	6.50 ± 0.14 **	50.64 ± 3.07 ****	1.71 ± 0.53 ^ns^	5.67 ± 0.45 ^ns^
Indomethacin	5.55 ± 0.04 ^ns^	57.88 ± 0.47 ****	2.04 ± 0.24 ^ns^	5.72 ± 1.34 ^ns^
AHA 300 mg/kg	6.35 ± 0.44 *	59.23 ± 1.45 ****	2.79 ± 1.29 ^ns^	5.05 ± 1.44 ^ns^
AHA 150 mg/kg	6.70 ± 0.66 **	51.34 ± 0.45 ****	1.93 ± 1.94 ^ns^	5.49 ± 1.49 ^ns^

**Table 7:** Liver Levels of Nitric Oxide (NO), Reduced Glutathione (GSH), Malondialdehyde (MDA), and Catalase (CAT). Values are expressed as mean ± SEM (n = 7). Statistical analysis was performed using one-way. * *p* < 0.05, ** *p* < 0.01, *** *p* < 0.001, **** *p* < 0.0001; ^ns^ indicates non-significant differences.

Group	GSH (nmole/mg Protein)	MDA (nmole/g Tissue)	NO (µM)	Cat (U/mg of Protein)
Arthritis control	16.15 ± 1.44	124.35 ± 1.02	53.16 ± 0.82	61.22 ± 6.59
Normal control	42.11 ± 4.88 ****	80.64 ± 3.88 ****	12.82 ± 3.13 ****	85.84 ± 1.00 **
Indomethacin	26.05 ± 0.002 *	93.65 ± 4.80 ***	16.77 ± 4.17 ****	71.67 ± 0.07 ^ns^
AHA 300 mg/kg	23.09 ± 0.56 ^ns^	85.07 ± 5.87 ****	16.00 ± 1.95 ****	75.66 ± 6.70 ^ns^
AHA 150 mg/kg	31.20 ± 2.31 ***	80.87 ± 5.44 ****	10.54 ± 2.26 ****	85.76 ± 6.29 **

**Table 8:** Spleen Levels of Nitric Oxide (NO), Reduced Glutathione (GSH), Malondialdehyde (MDA), and Catalase (CAT). Values are expressed as mean ± SEM (n = 7). Statistical analysis was performed using one-way. * *p* < 0.05, ** *p* < 0.01, **** *p* < 0.0001; ^ns^ indicates non-significant differences.

Group	GSH (nmole/mg Protein)	MDA (nmole/g Tissue)	NO (µM)	Cat (U/mg of Protein)
Arthritis control	23.78 ± 0.58	143.20 ± 4.77	54.96 ± 1.10	27.61 ± 3.39
Normal control	35.72 ± 2.93 **	82.56 ± 3.27 ****	21.84 ± 4.06 ****	40.19 ± 0.96 ^ns^
Indomethacin	30.15 ± 3.10 ^ns^	115.19 ± 4.80 *	27.67 ± 4.36 ****	39.55 ± 1.86 ^ns^
AHA 300 mg/kg	32.11 ± 2.93 ^ns^	109.21 ± 4.77 **	31.23 ± 1.22 ****	31.35 ± 1.17 ^ns^
AHA 150 mg/kg	33.78 ± 1.19 *	95.19 ± 11.07 ****	28.77 ± 1.48 ****	42.17 ± 7.12 *

## Data Availability

The original contributions presented in this study are included in the article. Further inquiries can be directed to the corresponding author.

## Supplementary Materials

The following supporting information can be downloaded at: https://www.mdpi.com/article/10.3390/plants15142164/s1, Figure S1: Images of nuclei obtained with a confocal fluorescence microscope, stained with ethidium bromide (5 μg/mL). (A) control (B) cells treated with 6 µg/mL of AHA (C) cells treated with 50 µg/mL of AHA (D) cells treated with 100 µg/mL of AHA.
