# Supplementary material for: Phytochemical Characterization and Evaluation of Antioxidant, Anti-Inflammatory, Cytotoxic, Genotoxic, and Anti-Arthritic Activities of Atriplex halimus Aqueous Leaf Extract
Source: Plants (Basel). 2026 Jul 14;15(14):2164. doi: 10.3390/plants15142164 (PMC13416023; doi:10.3390/plants15142164)
Supplement: Supplementary file 1 [file plants-15-02164-s001.zip › plants-4395158-supplementary.pdf]

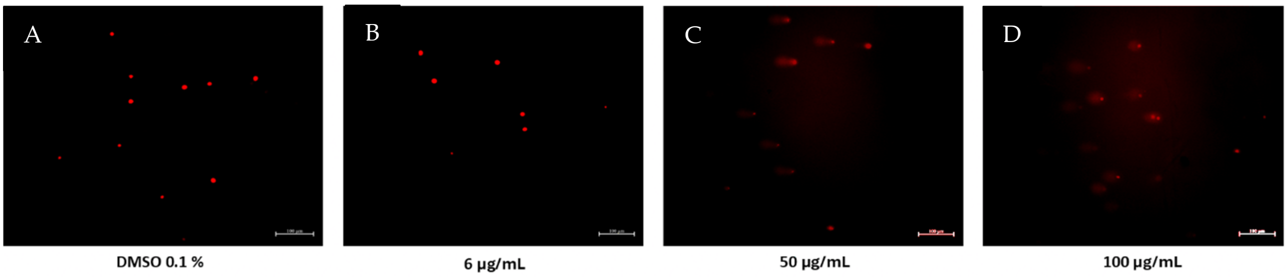

Figure S1. Images of nuclei obtained with a confocal fluorescence microscope, stained with ethidium bromide (5 µg/mL). (A) control (B) cells treated with 6 µg/mL of AHA (C) cells treated with 50 µg/mL of AHA (D) cells treated with 100 µg/mL of AHA.
